# The Plantaris Muscle Tendon and Its Relationship with the Achilles Tendinopathy

**DOI:** 10.1155/2018/9623579

**Published:** 2018-05-31

**Authors:** Ł. Olewnik, G. Wysiadecki, M. Podgórski, M. Polguj, M. Topol

**Affiliations:** ^1^Department of Normal and Clinical Anatomy, Medical University of Lodz, Poland; ^2^Department of Diagnostic Imaging, Polish Mother's Memorial Hospital Research Institute, Lodz, Poland; ^3^Department of Angiology, Medical University of Lodz, Poland

## Abstract

**Purpose:**

Although the plantaris muscle (PM) is vestigial in humans, it has a significant clinical role in procedures such as grafting. However, recent reports suggest its potential involvement in the tendinopathy of the midportion of the Achilles tendon. The aim of the study is therefore to evaluate morphological variation of the PM with regard to its potential conflict with the Achilles tendon.

**Material and Methods:**

Classical anatomical dissection was performed on 130 lower limbs (71 right, 59 left) fixed in 10% formalin solution. The morphology of the PM was assessed regarding the relationship between the course of the plantaris tendon and the calcaneal tendon.

**Results:**

The PM was present in 89.2% of cases. The findings indicate the presence of a new type of PM tendon insertion in which the tendon is inserted into the tarsal canal flexor retinaculum, potentially affecting the tendinopathy of the tibialis posterior muscle. In 26 cases (22.4%), insertion blended with the Achilles tendon (Type II), which may increase the risk of Achilles tendinopathy.

**Conclusion:**

The anatomical variation of PM tendon morphology may create a potential conflict with the Achilles tendon and the tibialis posterior tendon, thus increasing the possibility of tendinopathy.

## 1. Introduction

The plantaris muscle (PM) is typically characterized by a short, slim, and spindle-shaped muscle belly and long tendon [1–3]. The origin of the muscle is located on the popliteal surface of the femur above the lateral condyle and on the knee joint capsule [1]. The length of the muscle belly ranges from 50 to 100 mm and it is located between the popliteal muscle and the lateral head of the gastrocnemius muscle [1–3]. As it runs towards the medial crural region, the muscle belly becomes a long tendon. In its initial course, the tendon is located between the gastrocnemius muscle (GM) and soleus muscle (SM) [2–4], and upon leaving this section, in its distal course, the plantaris tendon is typically inserted into the calcaneal tendon [1]. Despite this classical schema, the course of the PM tendon, and especially its insertion, is characterized by high morphological variability [2, 5–9]. The plantaris muscle can be double [1, 10], and, in rare cases, it can be absent [3, 11–13].

An important clinical problem in recent years is Achilles tendon tendinopathy (ATT), which is difficult to cure [2, 3, 14]. Although ATT affects both physically active and inactive patients, it is more often associated with running or jumping disciplines [15, 16]: ATT is observed most commonly in runners (ultramarathons), tennis players, volleyball players, and football players. Despite recent advances, the pathogenesis of this disease remains not fully understood [15–18]. Recent years have seen an increase in interest in the possible involvement of the plantaris tendon in Achilles tendinopathy, and it is believed that the course of this tendon may affect the development of ATT [2, 3, 6–8, 16, 19].

The purpose of this study was to determine the anatomical relationship between the course of the plantaris tendon and the calcaneal tendon with regard to potential conflict associated with the tendons resulting in tendinopathy.

## 2. Material and Methods

In total, 130 lower limbs fixed in 10% formalin solution (71 right, 59 left) were obtained from adult cadavers. Consent for the study was given by the Local Bioethics Commission (agreement no. RNN/297/17/KE).

**Figure 1:**
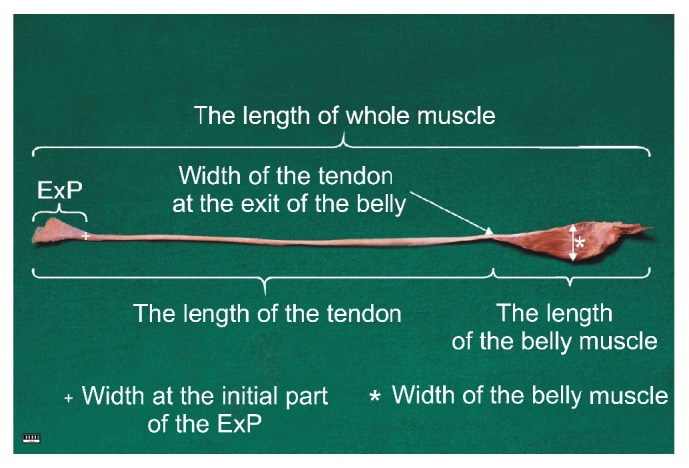
The basic measurements of the plantaris muscle.

A dissection of the crural region and foot area was performed using traditional techniques [2, 3, 20]. Upon dissection, the following morphological features of the PM were assessed:

- Relationship between the course of the plantaris tendon and the calcaneal tendon
- The location of the insertion of the plantaris tendon muscle
- Morphometric measurements (Figure 1)
- The characteristics of the extension point (ExP) [3] (width, thickness and distance between this point and the insertion of the plantaris tendon). ExP is the point at which the distal tendon begins to expand before its insertion [Figure 1]

An electronic digital caliper was used for all measurements (Mitutoyo Corporation, Kawasaki-shi, Kanagawa, Japan). Each measurement was carried out twice with an accuracy of up to 0.1 mm.

## 3. Statistical Analysis

The statistical analysis was performed using Statistica 12 software (StatSoft Polska, Cracow, Poland). A *p* value below 0.05 was considered significant. The results are presented as mean and standard deviation unless otherwise stated. The Chi^2^ test was used to compare the presence of PM between sexes and body sides. Continuous data was checked for normality with the Shapiro-Wilk test. As the data was not normally distributed, the Mann-Whitney* U* test was then used to compare the anthropometric measurements between the two types of PM course. The types of PM insertion were compared with regard to ExPs dimensions using the Kruskal-Willis ANOVA with dedicated post hoc tests. The correlation of continuous variables was assessed with Spearman's rank correlation coefficient.

## 4. Results

Our present findings serve as an extension and addition to the classification of previous study [3].

### 4.1. Frequency of Occurrence of the Plantaris Muscle

The PM was present in 116 lower limbs (89.2%) and absent on 14 limbs (10.8%). Although the absence of a PM is sometimes indicative that the muscle has become fused with the gastrocnemius or soleus muscle, no such condition was observed in the present sample.

The PM occurred in 51 (89.5%) men and 65 (89%) women, and in 64 (90.1%) cases on the right and 52 (88.1%) on the left limbs. Differences in occurrence between sexes and body sides were not statistically significant (*p* = 0.8367 and *p* = 0.9338, respectively).

### 4.2. Evaluation of Insertion of the Plantaris Tendon

The PM insertion was examined morphologically and classified according to the fivefold classification of Olewnik at al. [3].

**Figure 2:**
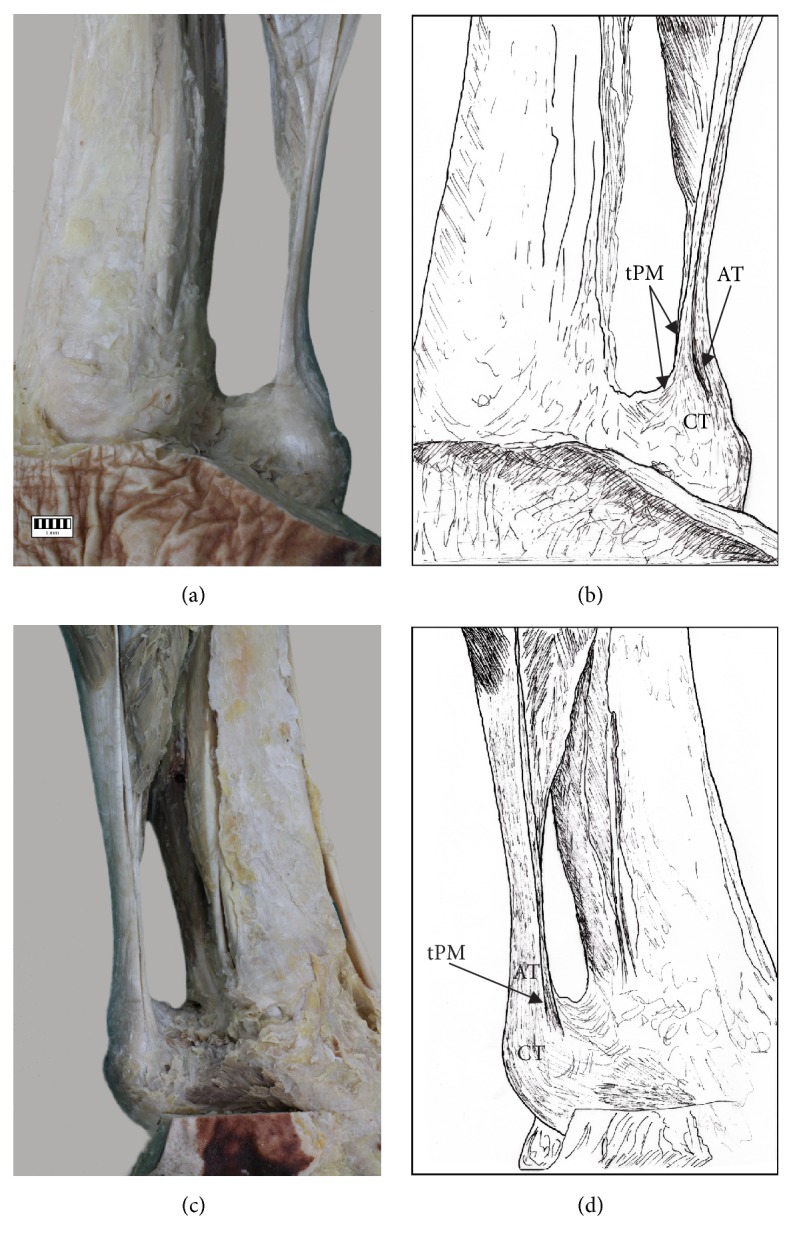
Types of insertion of the plantaris tendon: (a) Type I of insertion of plantaris tendon, (b) schema of Type I insertion (tPM: plantaris muscle tendon, AT: Achilles tendon, and CT: calcaneal tuberosity), (c) Type II plantaris tendon, and (d) schema of Type II insertion (PT: plantaris tendon, tPM: plantaris muscle tendon, AT: Achilles tendon, and CT: calcaneal tuberosity).

**Figure 3:**
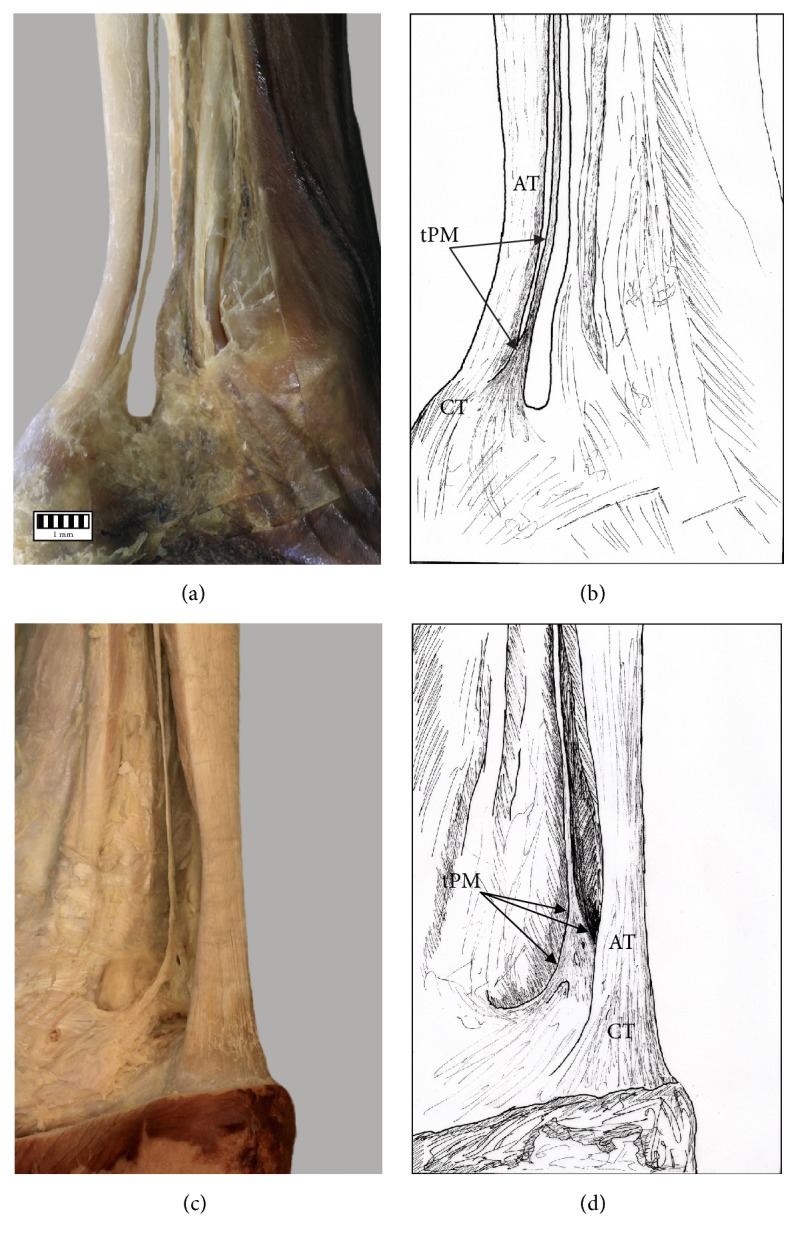
Types of insertion of the plantaris tendon: (a) Type III of insertion of plantaris tendon, (b) schema of the Type III of insertion (PT: plantaris tendon, tPM: plantaris muscle tendon, AT: Achilles tendon, and CT: calcaneal tuberosity), (c) Type IV of insertion of plantaris tendon, and (d) schema of the Type IV of insertion (PT: plantaris tendon, tPM: plantaris muscle tendon, AT: Achilles tendon, and CT: calcaneal tuberosity).

**Figure 4:**
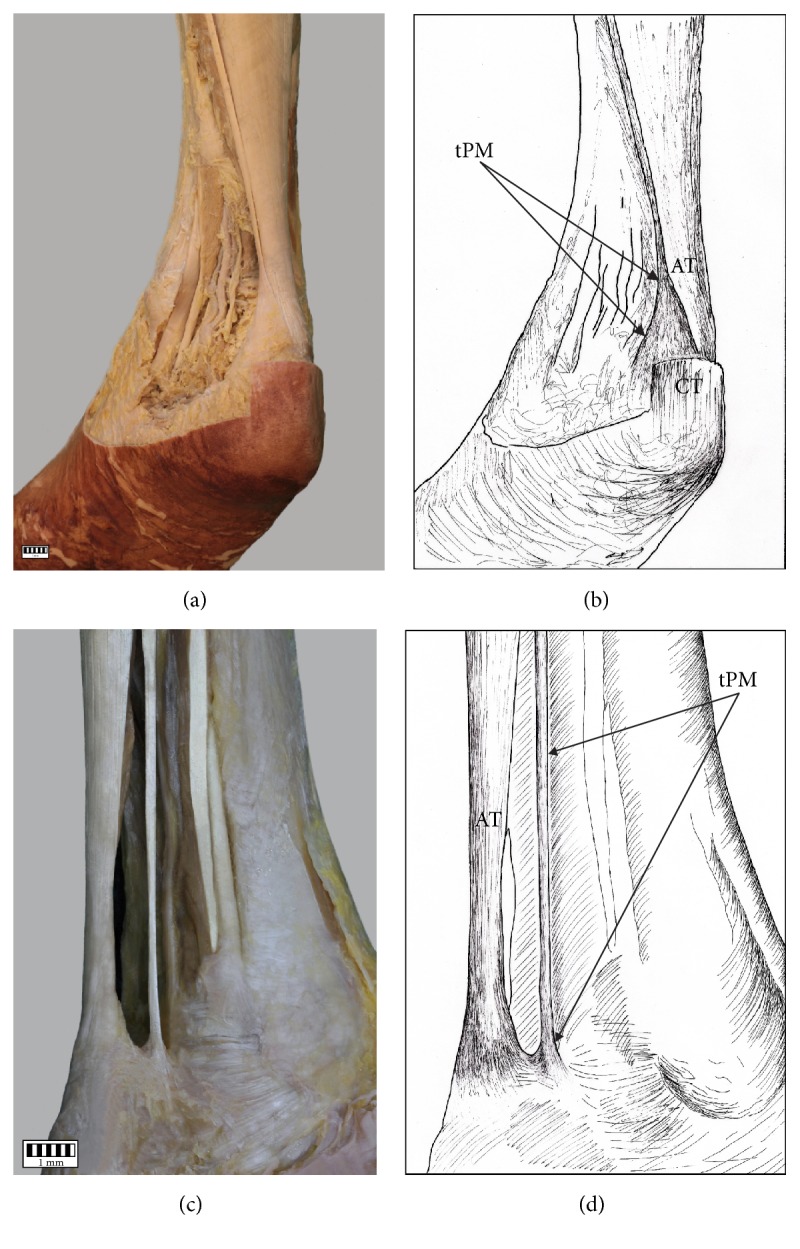
Types of insertion of the plantaris tendon: (a) Type V of insertion of the plantaris tendon, (b) schema of the Type V of insertion of the plantaris tendon muscle tPM tendon of plantaris muscle AT Achilles tendon CT calcaneal tuberosity, (c) Type VI of insertion of the plantaris tendon muscle, and (d) schema of the Type VI of insertion of the plantaris tendon tPM tendon of plantaris muscle AT Achilles tendon CT calcaneal tuberosity.

- Type I (51 cases, 44%) was characterized by a wide, fan-shaped insertion to the calcaneal tuberosity on the medial side of the calcaneal tendon (Figures 2(a) and 2(b)).
- Type II (26 cases, 22.4%) was characterized by insertion to the calcaneal tuberosity on the medial side, along with the Achilles tendon of the PT which was beaded in common parathendon with the calcaneal tendon (Figures 2(c) and 2(d)).
- Type III (8 cases, 6.9%) was characterized by insertion at the calcaneal bone, anterior to the calcaneal tendon (from 0.9 to 2.3 mm; Figures 3(a) and 3(b)).
- Type IV (4 cases, 3.4%) was characterized by the insertion to the deep crural fascia; the insertion was not located in the calcaneal bone. The PT has no direct “communication” with the calcaneal tendon, and the PT runs 2.3 to 2.4 mm anterior to it (Figures 3(c) and 3(d)).
- Type V (21 cases, 18.1%) was characterized by a very wide insertion encircling the posterior and medial surfaces of the calcaneal tendon (Figures 4(a) and 4(b)).

Additionally, six cases (5.2%) presented a type of insertion that has not been described before: one characterized with insertion at a point near to the tarsal canal flexor retinaculum of the leg (Figures 4(c) and 4(d)). This is proposed as a new Type VI.  Table 1 presents the morphological characteristics of ExPs in particular types of PM insertion.

The width of the ExP differed significantly between types of PM insertion (*p* < 0.0001) with Type I being significantly wider than Types II and VI; Type II being significantly narrower than Types I, IV and V; and Type VI being significantly narrower than Types I and V. The distance between the ExP and the PM insertion point also differed significantly (*p* = 0.0145), so that insertion was significantly closer to the calcaneus in Type VI than in Types IV and V. The thickness of the ExP did not differ significantly (*p* = 0.0524).

### 4.3. Evaluation of Variants of the Course of the Plantaris Tendon in relation to the Calcaneal Tendon

The course of the plantaris tendon was classified into two variants based on Olewnik et al. [3].

**Figure 5:**
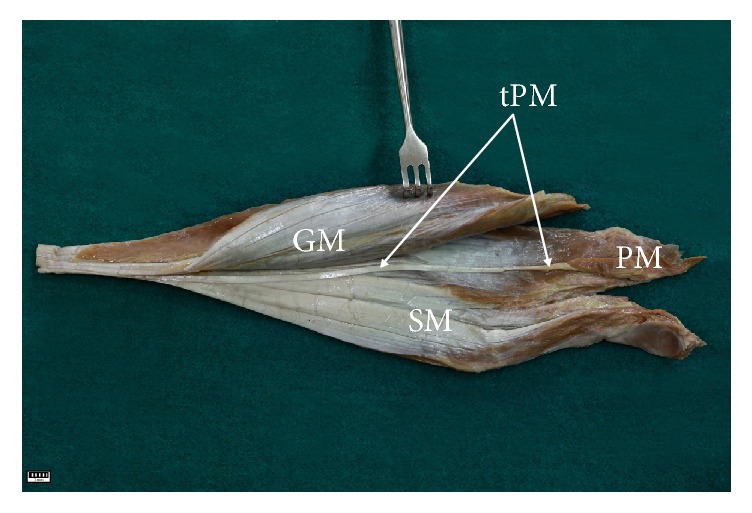
The space of the gastrocnemius muscle and soleus muscle. tPM tendon of the plantaris muscle PM plantaris muscle GM gastrocnemius muscle SM soleus muscle.

**Figure 6:**
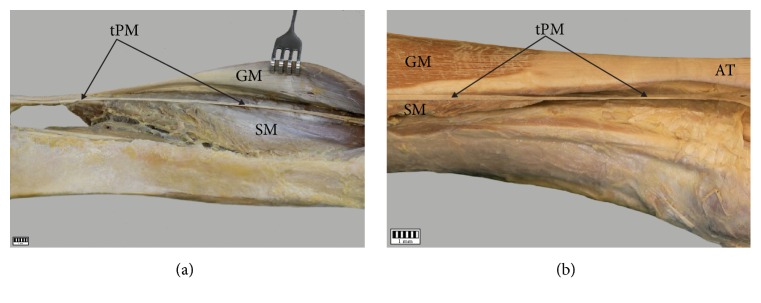
The variable anatomical correlation of the plantaris tendon. (a) Variant I of the plantaris tendon course. (b) Variant II of the plantaris tendon course. The plantaris tendon. GM gastrocnemius muscle, SM soleus muscle, and PT plantaris tendon.

In variant A (98 cases, 84.5%), the tendon was initially the space between the gastrocnemius muscle and soleus muscle (Figure 5) and then ran to the medial part of the leg; it was located on the medial side of the calcaneal tendon (Figure 6(a)).

In variant B (18 cases, 15.5%), the initial course resembled that of variant A; however, upon leaving the space between the GM and SM, it turned towards the medial crural region and ran directly anterior to the calcaneal tendon (Figure 6(b)). The characteristics of these two variants with regard to intention type are presented in Tables 2 and 3.

## 5. Discussion 

Increasing numbers of Achilles tendon disorders, including tendinopathy, are being recorded [21–25]. The midportion of the tendon is most commonly affected, accounting for 55–65% of Achilles tendon related pathologies, followed by insertional tendinopathy, accounting for 20–25% [23–26]. Midportion Achilles tendinopathy is very difficult to treat and its mechanism is not completely understood [2, 3, 6–9, 19, 25, 26].

Recent years have seen an increase in the number of studies on PM and its potential involvement in midportion Achilles tendinopathy [2, 3, 6–8, 16, 25–27].

Five types of insertion and two variants of PT course have previously been recognised [3]. Our findings extend this classification with Type VI, this being a PT that is inserted into the flexor retinaculum of the leg, and show that the individual types of insertion significantly differed from each other. Insertion classifications, including the one proposed in the present study, are presented in Table 4.

Interestingly, neither Cummins and Anson [27] nor Van Sterkenburg et al. [7] report the possibility of insertion to the flexor retinaculum of the leg: on rare occasions, this area is susceptible to tendinopathy and dislocation of the tibialis posterior muscle [28, 29]. It should be considered whether this type of insertion can predispose the patient to tendinopathy or dislocation of the tibialis posterior tendon.

Alfredson and Spang [25] note that Achilles midportion tendinopathy is more likely to affect men (65%) than women (35%). In addition, they found that, in 41% of patients, the plantaris tendon was located close to the medial side of the midportion of the Achilles tendon [9, 25]. In addition, van Sterkenburg et al. [8] note that the close connection between the calcaneal tendon and plantaris tendon was located at the level of the Achilles midportion tendinopathy. Alfredson [6] noted that patients complain of pain located between 2 and 7 cm above the calcaneal tuberosity on the medial side.

The findings of these anatomical and clinical studies suggest that the type of insertion and the course of the PM tendon can affect the occurrence of midportion tendinopathy. Variant A of tendon course and Type II of its insertion may predispose a patient to this condition, because the PT is beaded with common parathendon with Achilles tendon. Moreover, although it seems less probable, Types I, V, and VI, which are in close contact with the Achilles tendon, may also irritate/compress the Achilles tendon predisposing to tendinopathy.

In the present study, the PM was found to be absent in 14 lower limbs (10.8%). In these cases, the limb was carefully examined to confirm whether the PM had fused with the surrounding muscles. Harvey et al. [30] observed absence of the PM in 19% of cases, and Nayak et al. [31] in 7.69%. Simpson et al. [11] found this muscle to be absent between 7 and 20% of cases. Nevertheless, not all authors reported such an absence: Van Sterkenburg et al. [7] and Aragão et al. [12] note no cases of plantaris muscle absence, which begs the question of whether a lack of PM can have a significant effect on Achilles tendon tendinopathy. In our opinion, it cannot influence midportion Achilles tendinopathy, because there is no possibility of a relationship existing between variant A of PT course and Type II insertion. However, as PM tendon involvement is not fully understood, more clinical studies are required.

Ultrasound and colour Doppler examination have yielded reliable diagnoses of pathology within the calcaneal tendon and have proven valuable in evaluating morphological variation in this region [25, 32, 33]. As the rupture of the plantaris tendon may cause symptoms similar to deep vein thrombosis [13], ultrasound with a colour Doppler option seems to be the first line choice in planning surgery and diagnosing ruptures or deep vein thrombosis.

A limitation of this study is that it only speculates on the potential consequences of particular anatomical variants of PM tendon course and insertion. Nevertheless, it may serve as a starting point for further clinical studies including those including patients with tendinopathy.

## 6. Conclusion

Our findings indicate the presence of a new type of PM tendon insertion (Type VI) with a different potential role in tibialis posterior conflict. The course of the plantaris tendon and type of insertion may have a significant effect on the onset of Achilles midportion tendinopathy.

## Figures and Tables

**Table 1:** Morphological characteristics of ExP in different types of PM insertion.

PM tendon type (*n*)	Tendon width at the ExP mean (range) [mm]	Tendon thickness at the ExP mean (range) [mm]	Distance between ExP and insertion mean (range) [mm]
Type I (51)	3.73 (2.21–6.82)	1.22 (0.32–2.45)	34.33 (12.05–87.43)
Type II (26)	2.18 (1.12–4.05)	0.89 (0.37–1.73)	33.09 (10.26–61.51)
Type II (8)	2.74 (2.12–4.74)	1.27 (0.74–1.69)	38.67 (8.33–53.42)
Type IV (4)	4.06 (2.84–4.88)	0.72 (0.46–1.04)	54.26 (41.22–73.09)
Type V (21)	3.80 (2.19–6.12)	0.88 (0.32–1.29)	40.24 (20.89–49.68)
Type VI (6)	2.17 (1.27–3.21)	1.04 (0.46–1.76)	22.53 (18.69–33.21)

PM: plantaris muscle; ExP: extension point.

**Table 2:** Dependence between the insertion type and the plantaris tendon course variant.

Plantaris tendon course variant	Type of insertion of plantaris tendon
A	I, II, V
B	III, IV, VI

**Table 3:** Correlation between body side, gender and course variant of the PT.

	Right lower limb (*n* = 64)	Left lower limb (*n* = 52)	*p* value
COURSE VARIANT OF THE PLANTARIS TENDON			
Variant A [*n* (% null)]	55 (47.4)	43 (37.1)	0.8241
Variant B [*n* (% null)]	9 (7.8)	9 (7.8)	

	Men (*n* = 51)	Women (*n* = 65)	*p* value
COURSE VARIANT OF THE PLANTARIS TENDON			

Variant A [*n* (% null)]	43 (37.1)	55 (47.4)	0.8307
Variant B [*n* (% null)]	8 (6.9)	10 (8.6)	

**Table 4:** The differences between classifications given in the present study and those of other authors.

Types of insertion of the PtM	Cummins et al. [%]	Nayak et al. [%]	van Sterkenburg et al. [%]	Olewnik et al. [%]	Present [%]
Fan-shaped insertion into the medial calcaneal tuberosity	47	-	24	44	44

To the calcaneus, occurring 0.5–2.5 cm anterior to the medial boarder of the calcaneal tendon	36.5	-	1.8	8	6.9

Broad insertion investing the posteriori and medial surfaces on the adjacent distal calcaneal tendon	12.5	-	15	22	18.1

Insertion into the medial border of the calcaneal tendon at a level 1–16 cm proximal point at which the calcaneal tendon	4	-	-	-	-

insertion to the calcaneal tuberosity on the medial side, along with the Achilles tendon of the PT was in beaded in common parathendon with the calcaneal tendon	-	-	-	18	22.4

Insertion to the deep fascia	-	-	0.9	4	3.4

Insertion to the flexor retinaculum of the leg	-	28.8	-	-	5.2

Medial onto calcaneus	-	-	20	-	-

Medial onto calcaneal tendon	-	-	2.8	-	-

Medial with thin slips onto calcaneus	-	-	4.7	-	-

Anteromedial onto calcaneus	-	-	14	-	-

Anteromedial fan-shaped onto calcaneus	-	-	17	-	-

To the os calcaneus	-	36.5	-	-	-

To the calcaneal tendon at varus level	-	26.9	-	-	-

## Data Availability

Please contact authors for data requests (Ph.D. Ł. Olewnik, email address: lukasz.olewnik@umed.lodz.pl).
